# Evaluation for pharmacokinetic exposure of cytotoxic anticancer drugs in elderly patients receiving (R-)CHOP therapy

**DOI:** 10.1038/s41598-020-80706-2

**Published:** 2021-01-12

**Authors:** Junichi Nakagawa, Takenori Takahata, Rui Hyodo, Yu Chen, Kengo Hasui, Kota Sasaki, Kensuke Saito, Kayo Ueno, Kazuhiro Hosoi, Kazufumi Terui, Atsushi Sato, Takenori Niioka

**Affiliations:** 1 Department of Pharmacy, Hirosaki University Hospital, 53 Hon-cho, Hirosaki, Aomori 036-8563 Japan; 2 Department of Medical Oncology, Hirosaki University Graduate School of Medicine, 5 Zaifu-cho, Hirosaki, Aomori Japan; 3 Department of Pharmaceutical Science, Hirosaki University Graduate School of Medicine, 53 Hon-cho, Hirosaki, Aomori Japan

**Keywords:** Pharmacokinetics, Haematological cancer

## Abstract

(R-)miniCHOP therapy, which delivers approximately half-doses of the (R-)CHOP regimen, has shown efficacy and safety in patients who are more than 80 years old. This study aimed to compare the area under the plasma concentration–time curves (AUCs) of vincristine (VCR), doxorubicin (DXR), and cyclophosphamide (CPA) between (R-)CHOP and (R-)miniCHOP regimens. The AUCs were compared between patients aged 65–79 years receiving (R-)CHOP therapy and those aged 80 years and older receiving (R-)miniCHOP therapy. Age was not an independent variable for predicting the dose-adjusted AUCs (AUC/Ds) of cytotoxic anticancer drugs. The median AUCs of DXR and CPA were significantly smaller in the (R-)miniCHOP group than in the (R-)CHOP group (168.7 vs. 257.9 ng h/mL, *P* = 0.003, and 219.9 vs. 301.7 µg h/mL, *P* = 0.020, respectively). The median AUCs of VCR showed the same trend but the difference was not significant (24.83 vs. 34.85 ng h/mL, *P* = 0.135). It is possible that the AUCs of VCR, DXR, and CPA in patients aged 80 years and older receiving (R-)miniCHOP therapy may be lower than those in patients 65–79 years old receiving (R-)CHOP therapy.

## Introduction

Diffuse large B-cell lymphoma (DLBCL) accounts for more than 30% of all non-Hodgkin’s lymphoma (NHL) cases in Japan^1^, and its incidence continues to increase^2^. CHOP therapy, a multidrug combination chemotherapy with vincristine (VCR), doxorubicin (DXR), cyclophosphamide (CPA), and prednisolone (PSL), is the standard chemotherapy for the treatment of various subtypes of NHL, including DLBCL. In 2002, the anti-CD20 monoclonal antibody rituximab became available for use in combination with CHOP therapy (termed R-CHOP therapy), which results in a higher survival rate than CHOP therapy alone^3–5^. Presently, R-CHOP is the standard treatment for DLBCL, and this approach will likely continue for some time.

(R-)CHOP therapy has a variety of side effects, such as peripheral neuropathy, cardiac dysfunction, fever, and severe neutropenia^3^. Therefore, in clinical practice, dose reductions of cytotoxic anticancer drugs are occasionally required. Despite this, low relative or intended dose intensity (DI) reduces the efficacy of CHOP therapy and maintaining high treatment intensity is important for successful treatment of DLBCL^6–9^. These DIs may be associated with the pharmacokinetic exposure of cytotoxic anticancer drugs in CHOP therapy^7,9^. However, the minimum effective and toxic concentrations for each cytotoxic anticancer drug are not clear. In addition, it is unclear whether a dose adjustment based on body surface area (BSA) or age can be used uniformly to establish the pharmacokinetic exposure of these cytotoxic anticancer drugs.

The median age of DLBCL patients in Japan is 70 years old^1^, and the number of elderly patients with DLBCL is expected to increase along with the increasing size of the aging population. Elderly patients are generally known to be less tolerant to chemotherapy than adult patients^10^. In the last decade, reduced-dose (R-)CHOP regimens, such as (R-)miniCHOP, have been developed^11–13^. MiniCHOP therapy, which delivers approximate half-doses of the original CHOP regimen, has shown efficacy and safety in patients aged 80 years and older^11^. Since average global life expectancy has steadily increased, miniCHOP therapy might be useful for elderly patients with DLBCL. Previous studies assessed the treatment intensity of CHOP on the basis of a dose adjustment or an augmented treatment schedule^6–9^. However, the relationship between dose and blood concentration levels of cytotoxic anticancer drugs in elderly patients receiving CHOP therapy is not sufficiently clear.

In this study, we first investigated the factors that contribute to the effects of individual patient characteristics on the pharmacokinetics of VCR, DXR, and CPA in elderly patients aged 65 years or older. Next, we attempted to establish a simultaneous estimation method for area under the plasma concentration–time curves (AUCs) based on blood concentrations at the same sampling point to monitor the pharmacokinetic exposure of these cytotoxic anticancer drugs in the clinical setting. Finally, we compared the AUCs of VCR, DXR, and CPA between elderly patients aged 80 years and older receiving (R-)miniCHOP therapy and those aged 65–79 years old receiving (R-)CHOP therapy.

## Results

### Patient characteristics

Patient characteristics are listed in Table 1. A total of 20 patients (9 males and 11 females) were enrolled in this study. Eight patients were 80 years or older and receiving (R-)miniCHOP therapy. None of the patients had severe liver or renal dysfunction. There were no patients taking drugs that obviously affected the pharmacokinetics of VCR, DXR, or CPA, such as azole antifungal agents, cyclosporine, and drug-metabolizing enzyme-inducing antiepileptic drugs^14–17^. There were significant differences in median serum albumin (Alb) and estimated glomerular filtration rate (eGFR) between (R-)CHOP and (R-)miniCHOP groups (3.7 vs. 2.8 g/L, *P* = 0.047 and 70 vs. 47 mL/min, *P* = 0.020, respectively).

**Table 1:** Patient characteristics and administration dose of cytotoxic anticancer drugs.

	All patients (n = 20)	(R-)CHOP group (n = 12)	(R-)miniCHOP group (n = 8)	*P* values*
Sex (male:female)	9:11	6:6	3:5	0.465
Age (years)	76 ± 6.6 (67–92)	72 (70–73)	81 (80–85)	**–**
Body weight (kg)	57.6 ± 11.4 (38.5–77.8)	59.9 (54.8–69.0)	52.1 (42.9–62.3)	0.082
Body surface area (m^2^)	1.57 ± 0.18 (1.27–1.91)	1.66 (1.48–1.71)	1.51 (1.32–1.60)	0.181
AST (U/L)	30 ± 14 (15–65)	27 (24–36)	24 (20–39)	0.427
ALT (U/L)	25 ± 17 (10–68)	21 (16–50)	15 (11–22)	0.057
T-Bil (mg/dL)	0.5 ± 0.2 (0.2–0.9)	0.5 (0.4–0.7)	0.5 (0.4–0.6)	0.910
Alb (g/L)	3.3 ± 0.8 (1.8–4.5)	3.7 (3.0–4.3)	2.8 (2.6–3.2)	0.047
eGFR (mL/min)	65 ± 23 (45–131)	70 (53–87)	47 (44–63)	0.020
VCR (mg/body)	1.4 ± 0.3 (1.0–2.0)	1.6 (1.6–1.6)	1.0	**–**
DXR (mg/body)	53 ± 17 (30–95)	64 (55–65)	35 (30–40)	**–**
CPA (mg/body)	815 ± 231 (500–1400)	950 (850–1000)	600 (500–650)	**–**

Alb, serum albumin; ALT, alanine aminotransferase; AST, aspartate aminotransferase; CPA, cyclophosphamide; DXR, doxorubicin;

eGFR, estimated glomerular filtration rate; SD, standard deviation; T-Bil, serum total bilirubin; VCR, vincristine.

Values are presented as mean ± SD (range) or median (quartile 1–quartile 3).

*(R-)CHOP group vs. (R-)miniCHOP group.

### Pharmacokinetic analysis

#### Variation of pharmacokinetic parameters

**Figure 1:**
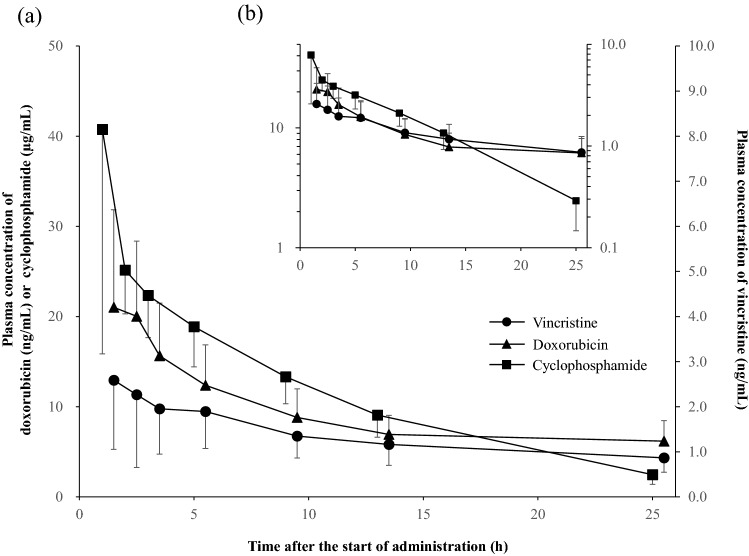
Concentration–time curve of cytotoxic anticancer drugs. (**a**) Linear- and (**b**) logarithmic-scale concentration–time curves of vincristine (circle), doxorubicin (triangle), and cyclophosphamide (square). Each point is presented as a mean ± standard deviation.

The pharmacokinetic profiles of each cytotoxic anticancer drug are shown in Fig. 1.

The variations of plasma concentration at each sampling point were larger for VCR and DXR than for CPA. The plasma concentrations of CPA and VCR after 9 and 9.5 h, respectively, from the start of administration generally decreased according to the first-order rate constant; the concentration of DXR showed a diphasic decrease.

Pharmacokinetic parameters of each cytotoxic anticancer drug are listed in Table 2. The coefficients of variation (%CV) of AUC and dose-adjusted AUCs (AUC/Ds) for all cytotoxic anticancer drugs were greater than 15% and those of VCR were the largest of the 3 agents (40.5% and 36.7%, respectively). The %CV of VCR t_1/2_ was larger than that of CPA; the %CV of DXR t_1/2_ could not be calculated because its concentration showed a diphasic decrease. Although the AUC of CPA was significantly correlated with the AUCs of DXR and VCR (*rho* = 0.549, *P* = 0.012, and *rho* = 0.550, *P* = 0.012, respectively), there was no significant correlation between the AUCs of DXR and VCR (*rho* = 0.096, *P* = 0.686).

**Table 2:** Pharmacokinetic parameters of cytotoxic anticancer drugs in all patients (n = 20).

Agents	Parameters	Unit	Mean ± SD	Median	Range	%CV
Vincristine	t_1/2_	(h)	30.2 ± 14.1	26.8	(14.0–61.6)	46.7
	AUC_1.5–25.5_	(ng h/mL)	32.0 ± 12.9	31.6	(11.8–70.9)	40.5
	AUC_1.5–25.5_/D	(ng h/mL/mg)	23.6 ± 8.7	23.1	(9.9–41.8)	36.7
Doxorubicin	t_1/2_	(h)	Not calculated			
	AUC_1.5–25.5_	(ng h/mL)	219 ± 69	206	(97–381)	31.6
	AUC_1.5–25.5_/D	(ng h/mL/mg)	4.3 ± 1.2	4.4	(1.9–6.7)	28.4
Cyclophosphamide	t_1/2_	(h)	6.5 ± 1.1	6.3	(4.5–8.3)	16.9
	AUC_1–25_	(µg h/mL)	276 ± 59	274	(184–386)	21.5
	AUC_1–25_/D	(µg h/mL/mg)	0.350 ± 0.069	0.330	(0.270–0.530)	19.6

AUC_n1–n2_, area under the concentration–time curve from n1 to n2 h;

AUC_n1–n2_/D, dose-adjusted area under the concentration–time curve from n1 to n2 h; t1/2, elimination half-life.

%CV, coefficient of variation.

#### Relationships between AUC/Ds and patient characteristics

The results of a univariate analysis of variance for AUC/D and t_1/2_ are shown in Table 3. Although there was no significant correlation between any of the patient variables and AUC/D of VCR, body weight and body surface area (BSA) had significant correlations with AUC/D of CPA and DXR (*P* < 0.05). Stepwise multiple linear regression analysis showed that the BSA and Alb were independent factors for predicting the AUC/D of DXR (partial *R*^2^ = 0.370, *P* = 0.001, and partial *R*^2^ = 0.307, *P* = 0.001, respectively), and BSA was an independent factor for predicting the AUC/D of CPA (*P* = 0.002).

**Table 3:** Correlations of dose-adjusted AUC and t_1/2_ with patient variables.

	Vincristine		Doxorubicin		Cyclophosphamide	
	AUC_1.5–25.5_/D	t_1/2_	AUC_1.5–25.5_/D	t_1/2_	AUC_1–25_/D	t_1/2_
	Median (ng h/mL/mg or h) of male and female (*P* values)					
Sex	23.6 vs. 23.1	30.1 vs. 30.4	3.99 vs. 4.75	–	310 vs. 370	6.83 vs. 6.18
	(0.882)	(0.766)	(0.230)		(0.016)	(0.175)
	*rho* (*P* values)					
Age	0.130	− 0.015	0.105	–	0.491	0.210
	(0.585)	(0.950)	(0.659)		(0.028)	(0.374)
Body weight	− 0.348	− 0.121	− 0.452	–	− 0.465	0.386
	(0.132)	(0.611)	(0.045)		(0.039)	(0.093)
BSA	− 0.291	− 0.120	− 0.486	–	− 0.587	0.369
	(0.213)	(0.613)	(0.030)		(0.007)	(0.110)
AST	− 0.078	0.467	0.050	–	0.006	0.198
	(0.745)	(0.038)	(0.833)		(0.980)	(0.402)
ALT	0.038	0.050	− 0.072	–	− 0.259	− 0.035
	(0.875)	(0.833)	(0.764)		(0.270)	(0.885)
T-Bil	0.061	0.015	− 0.326	–	− 0.311	0.152
	(0.799)	(0.950)	(0.160)		(0.182)	(0.521)
Alb	− 0.295	− 0.084	− 0.690	–	− 0.307	0.178
	(0.206)	(0.723)	(0.001)		(0.187)	(0.453)
eGFR	− 0.247	0.008	− 0.274	–	− 0.532	− 0.116
	(0.295)	(0.972)	(0.243)		(0.016)	(0.627)

Sex (male:female), 9:11.

Alb, serum albumin; ALT, alanine aminotransferase; AST, aspartate aminotransferase; AUC_n1–n2_/D, dose-adjusted area under the concentration–time curve from n1 to n2 h;

BSA, body surface area; eGFR, estimated glomerular filtration rate; T-Bil, serum total bilirubin; t_1/2_, elimination half-life.

#### Relationship between AUC and plasma concentrations at each sampling point

The correlations between AUC and plasma concentrations at each sampling point of VCR, DXR, and CPA are shown in Table 4. All plasma concentrations at each sampling point for these cytotoxic anticancer drugs were significantly correlated with AUC (all *P* < 0.01). The correlations between AUC_1–25 or 1.5–25.5_ and C_9 or 9.5_ for each cytotoxic anticancer drug were the best of all sampling points (all *rho* > 0.9, *P* < 0.001). The AUC estimation formulas using C_9–9.5_ for each cytotoxic anticancer drug were as follows: AUC_1.5–25.5 (VCR)_ = 25.16 · C_9.5_—2.01, *R*^2^ = 0.898, *P* < 0.001; AUC_1.5–25.5 (DXR)_ = 20.37 · C_9.5_ + 39.11, *R*^2^ = 0.867, *P* < 0.001; and AUC_1–25 (CPA)_ = 19.56 · C_9_ + 15.55, *R*^2^ = 0.972, *P* < 0.001.

**Table 4:** Correlations between AUC_1–25 or 1.5–25.5_ and plasma concentration at each sampling point for each cytotoxic anticancer drug.

	Vincristine	Doxorubicin	Cyclophosphamide
C_1 or 1.5_	0.945 (< 0.001)	0.895 (< 0.001)	0.576 (0.008)
C_2 or 2.5_	0.864 (< 0.001)	0.933 (< 0.001)	0.820 (< 0.001)
C_3 or3.5_	0.872 (< 0.001)	0.896 (< 0.001)	0.854 (< 0.001)
C_5 or 5.5_	0.921 (< 0.001)	0.901 (< 0.001)	0.861 (< 0.001)
C_9 or 9.5_	0.959 (< 0.001)	0.951 (< 0.001)	0.971 (< 0.001)
C_13 or 13.5_	0.929 (< 0.001)	0.908 (< 0.001)	0.915 (< 0.001)
C_25 or 25.5_	0.882 (< 0.001)	0.795 (< 0.001)	0.754 (< 0.001)

Values are expressed as Spearman’s *rho* values (*P* values). AUC_n1–n2_, area under the concentration–time curve from n1 to n2 h.

C_n_ represents plasma concentration of each anticancer drug at n hours after the start of administration.

#### Comparison of AUCs between (R-)miniCHOP and (R-)CHOP

**Figure 2:**
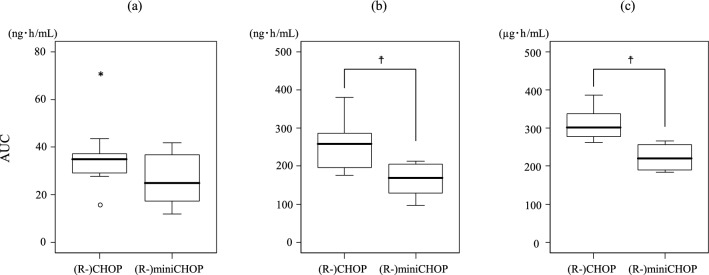
Comparison of AUC of cytotoxic anticancer drugs between (R-)CHOP and (R-)miniCHOP groups. **a** vincristine; **b** doxorubicin; **c** cyclophosphamide. The box spans data between 2 quartiles (interquartile range [IQR]), with the median represented as a bold horizontal line. The ends of the whiskers (vertical lines) represent the smallest and largest values that were not outliers. Outliers (circles) are values between 1.5 and 3 IQRs from the end of the box. Values more than 3 IQRs from the end of the box are defined as extreme (asterisk). AUC, area under the plasma concentration–time curve; AUC of vincristine and doxorubicin, AUC_1.5–25.5_; AUC of cyclophosphamide, AUC_1–25_; ^☨^*P* < 0.05.

The means ± standard devations of ratios of the actual dose to the dose of VCR, DXR, and CPA in the original (R)-CHOP or (R-)miniCHOP regimen were 82% ± 6% and 52% ± 3%, 79% ± 7% and 48% ± 3%, and 79% ± 6% and 52% ± 2%, respectively. Comparisons of the AUC of each cytotoxic anticancer drug between patients receiving (R-)miniCHOP and (R-)CHOP are shown in Fig. 2. The median AUCs of DXR and CPA were significantly lower in the (R-)miniCHOP group than in the (R-)CHOP group [(b) 168.7 vs. 257.9 ng h/mL, *P* = 0.003, and (c) 219.9 vs. 301.7 µg h/mL, *P* = 0.020, respectively]. The median AUC of VCR showed the same trend; however, the difference between the groups was not significant [(a) 24.83 vs. 34.85 ng h/mL, *P* = 0.135].

## Discussion

This study is the first report to show differences in AUCs of VCR, DXR, and CPA between (R-)CHOP and (R-)miniCHOP regimens. In addition, a simple estimation method of AUCs for these cytotoxic anticancer drugs was established. Until now, very few clinical studies have focused on the pharmacokinetics of cytotoxic anticancer drugs in elderly patients receiving CHOP therapy.

The AUCs of DXR and CPA were significantly lower in patients receiving (R-)miniCHOP therapy than in those receiving (R-)CHOP therapy. Although the AUC of VCR was not significantly different between the 2 groups, a similar trend was observed. Because the dose and dose range of VCR were lower and narrower than those of DXR and CPA, there may be no clear differences in these AUCs between groups. At this point, it remains unclear whether elderly patients aged 80 years and older should be treated with equal pharmacokinetic exposures of cytotoxic anticancer drugs as those for patients aged 65–79 years old. Still, it has been reported that the (R-)miniCHOP regimen provides a good balance between efficacy and safety in patients aged 80 years and older with DLBCL^9,11^. Therefore, the optimal pharmacokinetic exposure of cytotoxic anticancer drugs in these elderly patients may be lower than that of elderly patients aged 65 to 79 years old. Outcomes in elderly patients are worse than in younger patients, but numerous studies have shown that this difference cannot be explained by age alone^18,19^. Because it is important to complete the 6 cycles in CHOP therapy^20^, further investigations are required to determine whether the AUCs of cytotoxic anticancer drugs affect the relative DIs.

In this study, age was not an independent variable for predicting the AUC/Ds of VCR, DXR, and CPA in elderly patients aged 65 years and older receiving (R-)CHOP or (R-)miniCHOP therapy. Baudry et al. reported that aging did not affect the pharmacokinetics of DXR and CPA in very old patients (≥ 75 years old) receiving R-miniCHOP^21^. Our results agree with the findings of this study. BSA, which was an independent variable for predicting the AUC/Ds of DXR and CPA in this study, has been reported to affect pharmacokinetics parameters in other studies^22–24^. In addition, in this study, a negative correlation between Alb and AUC/D of DXR was found in elderly patients. It has been reported that DXR clearance (CL) correlates with various liver function test values, including Alb^25,26^. DXR is eliminated primarily by hepatic metabolism and biliary excretion. Although decreased Alb may represent decreased metabolic capacity, other liver function test values did not affect DXR AUC/D in this study. Further studies are needed to evaluate the relationship between Alb and pharmacokinetic parameters of DXR. Unlike with DXR and CPA, patient factors were not found to impact VCR AUC/D in this study. To the best of our knowledge, the effects of patient factors, such as sex, BSA, and biochemistry covariates, on VCR CL in elderly patients have not yet been clarified. Recently, it has been reported that gene polymorphisms of drug-metabolizing enzymes or transporters affect the pharmacokinetics of these cytotoxic anticancer drugs^23,27–30^. Thus, various factors have a potential impact on the pharmacokinetic exposure of elderly patients receiving CHOP therapy. However, these covariate factors alone could not fully explain individual variabilities in the pharmacokinetics of VCR, DXR, or CPA. Moreover, the associations between the AUCs of these cytotoxic anticancer drugs and the outcomes of patients with DLBCL are also unclear.

Plasma concentrations at 9–9.5 h after the start of administration were well correlated with AUCs within 24 h from the end of administration in patients receiving VCR, DXR, and CPA. Because accurate measurement of the AUC requires the collection and analysis of multiple blood samples, it is difficult to use this value as an indicator in the clinical setting. On the other hand, the simultaneous estimation method of AUCs of 3 cytotoxic anticancer drugs by a single blood collection at one sampling point is very convenient. The Bayesian estimation method provides a simple technique with good performance for estimating AUC of various drugs. Population pharmacokinetic parameters for DXR^21,31^ and CPA^21,32^ used in the Bayesian estimation method have already been reported. The plasma concentrations at 9–9.5 h after the start of administration are considered the best single sampling point for estimating AUCs of these cytotoxic anticancer drugs according to this Bayesian estimation method. Moreover, estimation of AUCs using a regression line is a simpler analytical method than Bayesian estimation, which requires the use of computer software for analysis. In the future, the AUC estimation formulas for VCR, DXR, and CPA constructed in this study may be useful for examining the relationships between pharmacokinetic exposure and relative DI in CHOP therapy in the clinical setting.

This study had a few limitations. First, the number of patients included in our study population was very small, so associations between AUCs and toxicities, such as severe myelosuppression and peripheral neuropathy, or efficacy, such as progression-free survival or overall survival, were not revealed. Therefore, pharmacokinetics–pharmacodynamics studies consisting of many elderly patients receiving (R-)CHOP or (R-)miniCHOP therapy are needed in the future. Second, the AUCs calculated in this study did not include area under the time during the infusion and the distribution phase, which has a rapid decrease of blood concentration immediately after the end of administration. However, because they cover most of the area of the elimination phase, they can be considered to be indicators of safety and effectiveness. Third, the AUC estimation formulas constructed in this study were derived from a very small number of patients receiving CHOP therapy; thus, validation studies in special populations, such as patients with liver dysfunction, low eGFR, or extreme BSA, are needed. However, our findings revealed that the AUCs of VCR, DXR, and CPA included in CHOP therapy can be simultaneously estimated by a single blood collection at one sampling point in the general elderly population. This AUC estimation method is therefore very simple. In conclusion, it is possible that the AUCs of VCR, DXR, and CPA in patients aged 80 years and older receiving (R-)miniCHOP therapy may be lower than those in patients 65–79 years old receiving (R-)CHOP therapy. However, the relationship between relative DIs and AUCs of cytotoxic anticancer drugs in elderly patients receiving CHOP therapy are not sufficiently clear. Simultaneous estimation of AUCs by a single blood collection at one sampling point may be useful for evaluating the pharmacokinetic exposure of these cytotoxic anticancer drugs in elderly patients receiving (R-)CHOP or (R-)miniCHOP therapy in the future.

## Methods

### Patients and protocols

**Figure 3:**
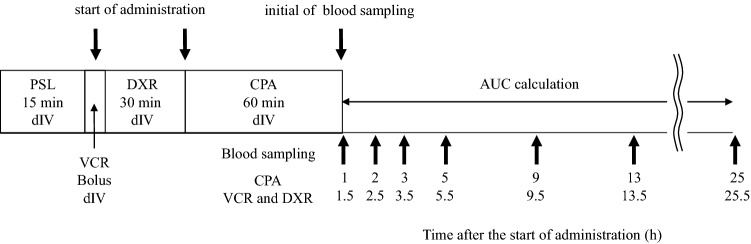
CHOP regimen and blood sampling protocol. AUC, area under the plasma concentration–time curve; CPA, cyclophosphamide; dIV, drip infusion into vein; DXR, doxorubicin; PSL, prednisolone; VCR, vincristine.

This study was carried out at a single institution. The study was conducted in accordance with the Declaration of Helsinki, and the protocol was approved by the Ethics Committee of Hirosaki University Graduate School of Medicine (project identification code: 2018-055). Patients receiving the first round of (R-)CHOP or (R-)miniCHOP therapy at Hirosaki University Hospital from October 2018 to April 2020 were enrolled in this study. Informed consent was obtained from all individual participants included in this study. The administration regimen for CHOP was as follows: PSL 100 mg intravenous (IV) infusion over 15 min on day 1, VCR 1.4 mg/m^2^ (maximum dose 2.0 mg) IV bolus over 5 min on day 1, DXR 35 mg/m^2^ IV infusion over 30 min on day 1, CPA 750 mg/m^2^ IV infusion over 1 h on day 1, and PSL 100 mg orally on days 2 through 5 (Fig. 3). Rituximab (375 mg/m^2^) was administered on a different day from CHOP day 1 administration. Approximately 20% dose reductions of VCR, DXR, and CPA were allowed at the discretion of the attending physician in the (R-)CHOP group. Patients who were 80 years and older received (R-)miniCHOP therapy (VCR 1 mg/body, DXR 25 mg/m^2^, CPA 400 mg/m^2^, and PSL 40 mg/m^2^). Plasma samples were centrifuged at 3500 rpm for 10 min at 4 °C and separated plasma was stored at − 80 °C until analysis.

### Dose intensity calculation

The DI of each cytotoxic anticancer drug was calculated as a percentage of the dose of the original CHOP regimen. For patients whose BSA exceeded 1.43 m^2^, the DI of VCR was calculated with 2 mg/body as 100%.

### Assay of plasma concentrations of VCR, DXR, and CPA

Plasma concentrations of cytotoxic anticancer drugs were measured by ultra-performance liquid chromatography tandem mass spectrometry (UPLC-MS/MS) using the ACQUITY UPLC System (Waters, MA, USA). Pretreatment of plasma samples and UPLC separation were performed based on the method we previously reported^33^.

Plasma (100 µL) was mixed with 150 µL of acetonitrile and 10 µL of internal standards (50 ng/mL trofosphamide for DXR and 20 µg/mL cyclophosphamide-d for CPA). The mixture was vortexed for 30 s and centrifuged at 13,500 rpm for 5 min at room temperature. Next, 100 µL of supernatant was diluted with 100 µL of MiliQ for DXR measurement, and 20 µL of supernatant was diluted with 380 µL of MiliQ for CPA measurement. Samples for VCR measurement were prepared according to a different procedure than those for DXR and CPA measurement. Plasma (200 µL) was mixed with 300 µL of acetonitrile and 20 µL of internal standards (50 ng/mL vinblastine [VBL]). The mixture was vortexed for 30 s and centrifuged at 13,500 rpm for 5 min at room temperature. Next, 350 µL of supernatant was evaporated using the centrifuge evaporator and then redissolved with 100 µL of 30% acetonitrile. The mixture was vortexed for 30 s and centrifuged at 13,500 rpm for 1 min at room temperature. The sample was transferred to an autosampler vial and then 2 µL for CPA, 8 µL for DXR, and 10 µL for VCR were injected into an ACQUITY UPLC HSS C18 column (1.7 um, 100 mm × 2.1 mm) at 40 °C. The mobile phase consisted of (A) MiliQ with 0.1% formic acid and (B) acetonitrile with 0.1% formic acid at a flow rate of 0.4 mL/min. Gradient conditions were as follows: 0–1.0 min, held in 5% B; 1.0–6.0 min, linear from 5 to 95% B; 6.0–7.0 min, held in 95% B; 7.0–7.1 min, linear from 95 to 5% B; 7.1–10.0 min, held in 5% B. The analyte and internal standard were ionized and detected using Xevo TQD (Waters, MA, USA). Positive electrospray ionization was performed in the multiple reaction monitoring mode. Ion transitions were as follows: m/z 413.5 → 353.1 for VCR, 544.2 → 397.0 for DXR, and 265.1 → 140.0 for CPA; internal standards were m/z 811.4 → 751.2 for VBL, 323.0 → 154.0 for trofosphamide, and 265.1 → 140.0 for cyclophosphamide-d. Cone voltage and collision energies were 60 V and 40 eV for VCR and VBL, 30 V and 20 eV for CPA and cyclophosphamide-d, 30 V and 10 eV for DXR, and 30 V and 30 eV for trofosphamide, respectively. The lower limits of quantitation for VCR, DXR, and CPA were 0.5 ng/mL, 1 ng/mL, and 1 µg/mL, respectively. The calibration curve was linear in the following ranges: VCR, 0.5–10 ng/mL; DXR, 2–80 ng/mL; CPA, 2.5–40 µg/mL. If the plasma concentration of CPA exceeded the upper limit of the standard curve, the sample was diluted by one-third. The intra- and inter-day accuracy values (CV%) were all within ± 15% and precision values (CV%) were all less than 15% in each calibration curve range.

### Pharmacokinetic analysis

Pharmacokinetic analyses with VCR, DXR, and CPA were carried out using a standard non-compartmental model with Phoenix WinNonlin (Pharsight Co, Mountain View, CA, version 8.1). Plasma samples of CPA, (VCR) and (DXR) were collected at 1 (1.5), 2 (2.5), 3 (3.5), 5 (5.5), 9 (9.5), 13 (13.5) and 25 (25.5) h after the start of each drug administration. The AUCs of each cytotoxic anticancer drug were calculated on the basis of plasma concentrations after initial blood sampling (Fig. 3). Each AUC was calculated using the linear trapezoidal rule. The elimination half-life (t_1/2_) was obtained using the log-linear regression of the terminal phase of the concentration–time data for at least 3 sampling points (elimination half-life = ln2/k_e_; k_e_ = elimination rate constant).

### Statistical procedures

The Shapiro–Wilk test was used to assess distribution. The characteristics of patients and pharmacokinetic parameters of VCR, DXR, and CPA are expressed as means ± standard deviation or medians and range. The Mann–Whitney U test was used to determine the difference in continuous values between groups. Spearman’s rank correlation coefficient test was used to assess correlations in continuous values between groups, and all results are expressed as Spearman’s *rho* values. Factors with *P-*values less than 0.05 in a univariate analysis were included in a stepwise multiple linear regression analysis to identify independent factors for predicting results. The estimation formulas were constructed using simple linear regression analysis. The percent variation that could be explained by the simple regression equation was expressed as a coefficient of determination (*R*^2^). A *P *value less than 0.05 was considered statistically significant. Statistical analysis was performed with SPSS 26.0 for Windows (SPSS IBM Japan Inc., Tokyo, Japan).

## Data Availability

The datasets used and/or analyzed during the current study are available from the corresponding author on reasonable request.
